# Impact of Global Sagittal Spinal Alignment on Degenerative Lumbar Scoliosis

**DOI:** 10.7759/cureus.83612

**Published:** 2025-05-06

**Authors:** Hideya Yamauchi, Kenji Endo, Yasunobu Sawaji, Hirosuke Nishimura, Kengo Yamamoto

**Affiliations:** 1 Orthopedic Surgery, Tokyo Medical University, Tokyo, JPN

**Keywords:** coronal spine alignment, degenerative lumbar scoliosis, diagnosis, lumbar spinal canal stenosis, pelvic incidence, sagittal spine alignment

## Abstract

Background: In spine surgery, understanding the balance between sagittal and coronal planes, taking both spinal alignment and pelvic orientation into account, is crucial. The purpose of this study was to clarify the influence of pelvic incidence (PI) on spinopelvic parameters in patients with degenerative lumbar scoliosis (DLS) by comparing them to those without DLS.

Method: The subjects were 259 patients (146 men and 113 women, mean age 69.4 years) who underwent surgery in our department between January 2010 and August 2018. The nonscoliosis group (N group, Cobb angle: 0°-9°; n = 161) and the scoliosis group (S group, Cobb angle: 10°-29°; n = 98) were used to compare their spinal alignments.

Result: Regarding the parameters of sagittal spinal alignment, lumbar lordosis (LL) (N group 35.3 ± 12.5°; S group 31.6 ± 14.9°) was significantly smaller (p < 0.05) and PI (N group 46.6 ± 11.6°; S group 52.3 ± 12.1°) and PI-LL (N group 11.8 ± 14.3°; S group 21.0 ± 17.5°) were significantly larger (p < 0.001) in the S group than in the N group. Positive correlations were observed between Cobb angle and sagittal vertical axis (SVA), pelvic tilt (PT), PI, and PI-LL, and a negative correlation was observed between Cobb angle and LL.

Conclusion: The incidence of DLS in middle-aged and older patients is related to PI, and the coronal Cobb angle is positively correlated with PI and PI-LL and is negatively correlated with LL. Coronal deformity could be affected by both pelvic orientation and sagittal spine alignment.

## Introduction

Degenerative lumbar scoliosis (DLS) is associated with degeneration of the intervertebral disc, vertebral body, and facet joint, and it typically occurs in people over the age of 50 [1]. DLS is a three-dimensional deformity commonly presenting as kyphoscoliosis, in which scoliosis featuring vertebral wedging and lateral slipping is accompanied by kyphosis [2,3]. Factors associated with the progression of DLS have been reported in a systemic review showing that the progression of intervertebral disc degeneration, an intercrest line through L5, and lateral vertebral translation more than 6 mm are associated with the progression of DLS [4].　However, the association between sagittal plane misalignment (pelvic posterior tilt) and curve progression still needs to be investigated. The progression of scoliosis is reported to lead to a decrease in lumbar lordosis (LL), thereby resulting in pelvic verticalization [5,6]. Therefore, a coronal deformity is considered to be affected by both the pelvic orientation and sagittal spinal alignment.

Understanding of sagittal and coronal balance with consideration of both spinal alignment and pelvic orientation is indispensable in order for spine surgeons to achieve successful outcomes from spinal operative procedures. In particular, the correlation between pelvic orientation and degenerative scoliosis is important for the prediction of scoliosis progression. Pelvic incidence (PI) is a pelvic morphologic angle unique to each individual and is not influenced by posture, and PI determines the sagittal alignment and affects the compensatory function of the lumbar spine [7]. Therefore, large PI and PI-LL mismatches have been reported to associate with poor outcome and poor postoperative symptom improvement rates in lumbar interbody fusion surgery in DLS [8,9], and PI and PI-LL may also be important parameters in surgical planning for DLS, such as decompression, fixation, or three-dimensional alignment correction, depending on the pathological condition [10,11]. The purpose of this study is to clarify the influence of PI on spinopelvic parameters in patients with DLS by comparing them to those without DLS.

## Materials and methods

The subjects were consecutive 259 patients (146 men and 113 women, mean age 69.4 years) who underwent surgery in our department between January 2010 and August 2018. Patients with vertebral body fracture, history of spinal surgery, rotational deformity, or severe scoliosis with a Cobb angle over 30° were excluded [12].

**Figure 1:**
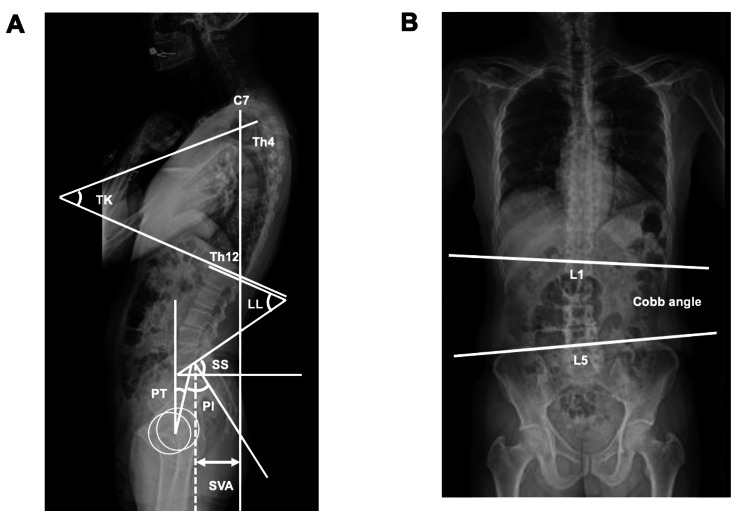
Measurements of radiological parameters Lateral (A) and anteroposterior (B) standing radiographs SVA: sagittal vertical axis; TK: thoracic kyphosis angle; LL: lumbar lordosis; SS: sacral slope; PT: pelvic tilt; PI: pelvic incidence; Cobb angle: between the inferior endplates of L1 and L5

We assessed preoperative sagittal alignment, including lumbopelvic sagittal alignment. The parameters measured were the thoracolumbar Cobb angle from frontal full-length radiographs of the spine and the distance from the C7 plumb line to the posterior-superior corner of the sacrum (sagittal vertical axis; SVA), thoracic kyphosis angle (TK, T4-T12), lumbar lordotic angle (LL, L1-S1), pelvic tilt (PT), and PI from lateral full-length radiographs of the spine (Figure 1A and Table 1). The subjects were divided into the nonscoliosis group (N group, Cobb angle: 0°-9°) and the scoliosis group (S group, Cobb angle: 10°-29°) to compare spinal alignment between the two groups (Figure 1B and Table 1).

**Table 1:** Measurement method of sagittal spinal parameters

Measurement site	Measurement method
TK: thoracic kyphosis angle	Angle from the upper endplate of T4 to the lower endplate of T12
LL: lumbar lordosis angle	Angle from the upper endplate of L1 to the upper sacral endplate
PT: pelvic tilt angle	Angle between the line connecting the midpoint of the sacral plate to the axis of the femoral heads and the gravity line
SS: sacral slope	Angle between the tangent to the upper edge of the sacrum and the horizontal plane
PI: pelvic incidence	Angle between a line perpendicular to the sacral plate at its midpoint and a line connecting the same point to the center of the bicoxofemoral axis
SVA: sagittal vertical axis	Length from the plumb line dropped from the posterior-superior corner of the S1 vertebral body to the plumb line dropped from the center of C7

Data were obtained using the digital slot-scanning radiography mode of the Sonialvision Safire fluoroscopy system (Shimadzu Corporation, Kyoto, Japan). The intra- and inter-observer agreements of the measurement were described in detail in a previous paper [13]. This study was conducted with the approval of the University Research Ethics Committee.

For statistical analysis, an unpaired t-test was performed using JMP® version 12 (SAS Institute Inc., Cary, NC, USA) to compare the two groups. The Pearson product-moment correlation coefficient was calculated to evaluate the relationship between parameters, and a significance level of less than 5% (p < 0.05) was considered to indicate a statistically significant difference.

## Results

The N group consisted of 161 cases (male: 59 cases; female: 102 cases; average age: 68.5 ± 10.8 years) and the S group 98 cases (male: 44 cases; female: 54 cases; average age: 71.6 ± 9.4 years). There was no statistically significant gender difference between the N and S groups, but age was significantly higher in the S group (p < 0.05). Regarding the parameters of sagittal spinal alignment, LL (N group, 35.3 ± 12.5°; S group, 31.6 ± 14.9°) was significantly smaller (p < 0.05), and PI (N group, 46.6±11.6°; S group 52.3 ± 12.1°) and PI-LL were significantly larger (p < 0.001) in the S group than in the N group (Table 2).

**Table 2:** Comparison of sagittal spinal aliment parameters between nonscoliosis and scoliosis groups Values are expressed as mean ± standard deviation. N group: nonscoliosis group; S group: scoliosis group; SVA: sagittal vertical axis; TK: thoracic kyphosis angle; LL: lumbar lordotic angle; SS: sacral slope; PT: pelvic tilt; PI: pelvic incidence

	N group (n = 161)	S group (n = 98)	p-value
Cobb angle (degrees)	3.4±3.3	15.7±5.0	*<0.0001
SVA (mm)	49.1 ± 43.1	52.8 ± 46.6	0.54
TK (degrees)	29.2 ± 11.4	28.9 ± 13.3	0.88
LL (degrees)	35.3 ± 12.5	31.6 ± 14.9	*0.04
SS (degrees)	28.6 ± 8.3	29.5 ± 8.9	0.42
PT (degrees)	21.3 ± 23.5	29.5 ± 8.9	0.12
PI (degrees)	46.6 ± 11.6	52.3 ± 12.1	*0.0005
PI-LL (degrees)	11.8±14.3	21.0±17.5	*<0.0001

Regarding the correlation between the coronal plane and the sagittal plane parameters in the entire study population, a positive correlation was observed between Cobb angle and SVA, PT, PI, and PI-LL, and a negative correlation was noted between Cobb angle and LL (Table 3). 

**Table 3:** Correlation of Cobb angle between spinal sagittal alignment parameters SVA: sagittal vertical axis; TK: thoracic kyphosis angle; LL: lumbar lordotic angle; SS: sacral slope; PT: pelvic tilt; PI: pelvic incidence; r = correlation coefficient; *: significant correlation (p < 0.05)

	Vs Cobb angle (r value)	Vs Cobb angle (p-value)
SVA (mm)	0.15	0.018*
TK (degrees)	-0.05	0.44
LL (degrees)	-0.19	0.0041*
SS (degrees)	0.0009	0.99
PT (degrees)	0.19	0.0037*
PI (degrees)	0.23	0.0005*
PI-LL (degrees)	0.33	<0.0001*

**Figure 2:**
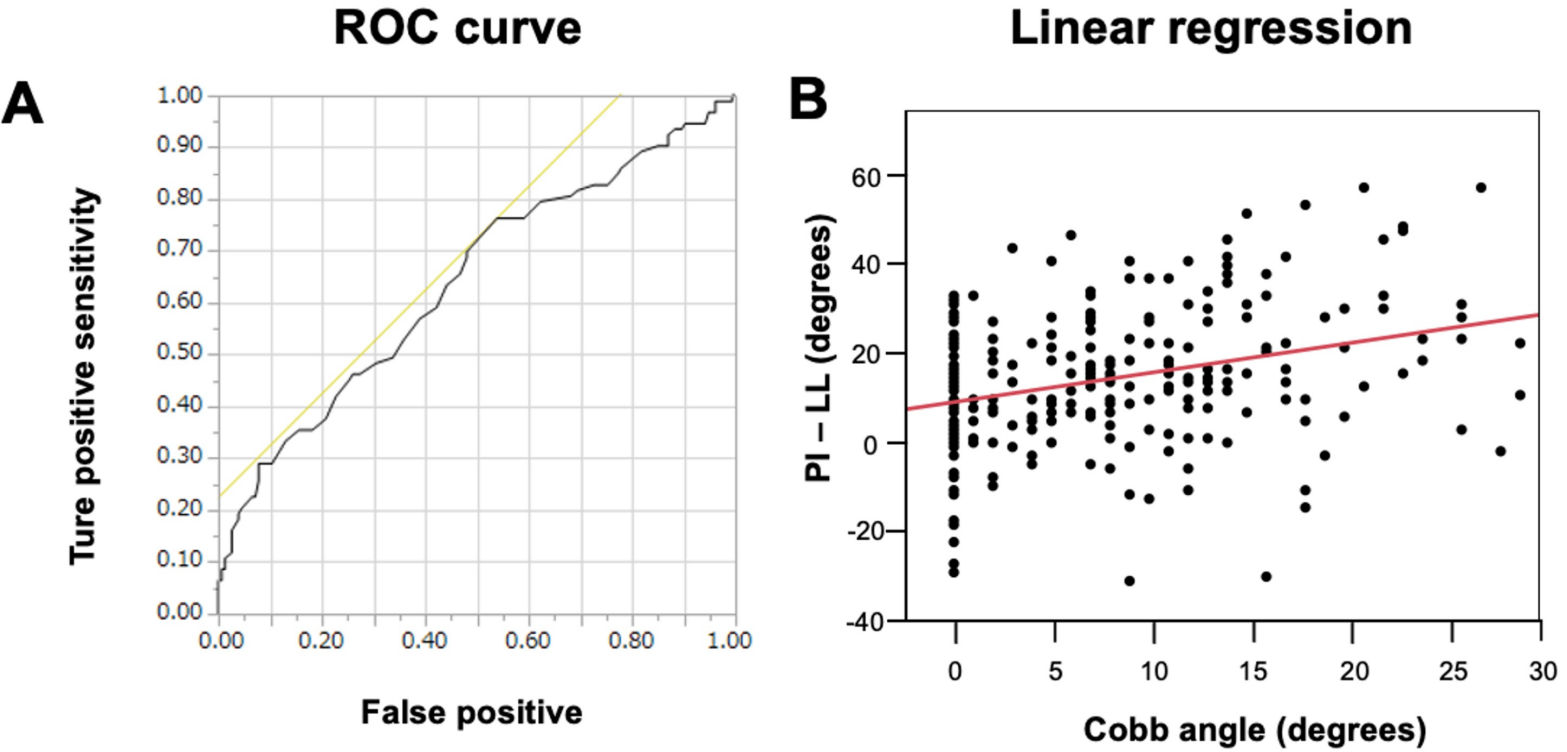
Visual representation of the regression models superimposed on raw data (A) ROC curve. (B) Linear regression. A positive correlation was observed between Cobb angle and PI-LL. AUC = 0.64 (95%CI 0.57-0.71). The cut-off values of PI-LL of the ROC curve and linear regression between the scoliosis group and nonscoliosis group were 11° (ROC curve) and 17° (Linear regression), respectively. ROC: receiver operating characteristic, PI: pelvic incidence, LL: lumbar lordosis

The cut-off values of PI-LL between the scoliosis and nonscoliosis groups were 11° (receiver-operating characteristic (ROC) curve) and 17° (linear regression) (Figure 2).

## Discussion

A large PI is reported to be associated with the onset of degenerative spondylolisthesis in the sagittal plane [14] and the onset of hip osteoarthritis [15]. However, only a few studies have reported that a large PI is involved in the coronal plane balance [16,17].

Regarding the whole-spine alignment in DLS, some studies have reported that LL is decreased with increasing Cobb angle and that once the compensatory capacity of the pelvis is exceeded, anterior tilt of the trunk occurs [2,3]. In the present study, we also observed a significant decrease in LL in the scoliosis group, and LL showed a decreasing tendency, and SVA showed an increasing tendency as the Cobb angle increased. The Cobb angle was positively correlated with PI and PI-LL, which may suggest that cases with large PI-LL tend to progress the decrease in LL and scoliosis; conversely, small PI-LL would be a background to limit the progression of scoliosis in DLS. A large PI-LL may signify the degeneration of the middle and lower intervertebral discs in the lumbar spine and a decrease in LL. This may contribute to the development of scoliosis by making it impossible to maintain LL to correspond to the PI [18]. Anteversion of the trunk is also reported to be involved in the development of scoliosis, although details such as the mechanism of development are still unknown [18].

When lumbar disc degeneration occurs due to aging, the rotation angle of the vertebral body tends to increase, and asymmetric degeneration is likely to occur [6]. Asymmetric degeneration of the intervertebral disc has been reported to be involved in the progression of scoliosis [19]. PI-LL mismatch is more likely to occur in cases with a large PI followed by a decrease in LL due to disc degeneration. Taken together, it would be possible to speculate that a positive correlation between PI and Cobb angle was observed in this study.

Large PI is thought to promote the progression of degenerative scoliosis by the following mechanism. Previous reports based on cadavers suggested that forces in the anterior and posterior directions in the lumbar spine induce lateral movement forces [18,20], which may contribute to the asymmetrical degeneration of the intervertebral discs. Another study reported that in patients having a large PI, anterior-posterior movements of the pelvis and lumbar spine are increased [21], which is likely to induce lateral movements. Forces from lateral movements cause asymmetric disc degeneration, possibly playing a role in the progression of degenerative scoliosis. Previous reports have stated that large PI causes changes in stress distribution, leading to uneven degeneration of the intervertebral discs, which in turn causes scoliosis [17].

Considering the results in the present study and clinical settings, in neurological symptoms including pain and numbness in the lower limb of patients with lumbar canal stenosis (LCS), the scoliosis progression may worsen those symptoms by the lateral intervertebral foraminal stenosis progression [22]. When those patients have large PI and PI-LL mismatch, careful observation will be required for potential progression of scoliosis [5,23]. Our findings suggested an increased risk of progression of degenerative scoliosis in patients with large PI and PI-LL. In the future, these results could help to predict the prognosis of lumbar spinal canal stenosis complicated by DLS and may serve as useful information for the selection of decompression, fusion, or alignment correction as a surgical method. For example, DLS patients having large PI and PI-LL mismatch may require a corrective surgery to obtain appropriate LL to match the individual PI to manage scoliosis progression.

At the radiographic parameter threshold predictive of an Oswestry Disability Index (ODI) score of 40, PI-LL had a value of 11° [12] (Cobb angles of 30° or more were included). In the present study, the cut-off value of PI-LL between the S and N groups was similar to this value. It is indicated that a coronal deformity could be affected by both pelvic orientation and sagittal spine alignment. This may be useful information for the selection of decompression, fixation, and alignment correction as surgical treatments.

Limitations of this study include the following: compensatory function of the lower limbs in sagittal alignment was not evaluated; due to retrospective cross-sectional study design, longitudinal analysis could not be performed, but it needs to be investigated in future studies; cases with Cobb angle more that 30° were not analyzed; only surgical cases were examined; the correlation coefficient between the Cobb angle and PI/PI-LL was low, which may imply the involvement of multiple factors including the height of the L5 disc and the iliac crest, sex, race, work environment and lifestyle [24]; relationships with clinical symptoms were not investigated.

## Conclusions

The incidence of DLS in middle-aged and older patients was related to PI. The coronal Cobb angle was positively correlated with PI and PI-LL and was negatively correlated with LL. Coronal deformity could be affected by both pelvic orientation and sagittal spine alignment. Spinal malalignment needs to receive attention when planning a surgical decision for patients with large PI and PI-LL mismatch among DLS patients.
